# Aspirin‐Mediated Acetylation of SIRT1 Maintains Intestinal Immune Homeostasis

**DOI:** 10.1002/advs.202306378

**Published:** 2024-03-14

**Authors:** Liangguo Xie, Chaoqun Li, Chao Wang, Zhen Wu, Changchun Wang, Chunyu Chen, Xiaojian Chen, Dejian Zhou, Qiang Zhou, Ping Lu, Chen Ding, Chen‐Ying Liu, Jinzhong Lin, Xumin Zhang, Xiaofei Yu, Wei Yu

**Affiliations:** ^1^ State Key Laboratory of Genetic Engineering School of Life Sciences Zhongshan Hospital Fudan University Shanghai China; ^2^ Department of Colorectal and Anal Surgery Xinhua Hospital Shanghai Jiao Tong University School of Medicine Shanghai China; ^3^ Department of Research Center for Molecular Recognition and Synthesis Department of Chemistry Fudan University Shanghai China

**Keywords:** acetylation modification, apoptosis, aspirin, intestinal immune homeostasis, SIRT1 deacetylase

## Abstract

Aspirin, also named acetylsalicylate, can directly acetylate the side‐chain of lysine in protein, which leads to the possibility of unexplained drug effects. Here, the study used isotopic‐labeling aspirin‐d^3^ with mass spectrometry analysis to discover that aspirin directly acetylates 10 HDACs proteins, including SIRT1, the most studied NAD^+^‐dependent deacetylase. SIRT1 is also acetylated by aspirin in vitro. It is also identified that aspirin directly acetylates lysine 408 of SIRT1, which abolishes SIRT1 deacetylation activity by impairing the substrates binding affinity. Interestingly, the lysine 408 of SIRT1 can be acetylated by CBP acetyltransferase in cells without aspirin supplement. Aspirin can inhibit SIRT1 to increase the levels of acetylated p53 and promote p53‐dependent apoptosis. Moreover, the knock‐in mice of the acetylation‐mimic mutant of SIRT1 show the decreased production of pro‐inflammatory cytokines and maintain intestinal immune homeostasis. The study indicates the importance of the acetylated internal functional site of SIRT1 in maintaining intestinal immune homeostasis.

## Graphical abstract

Using isotopic labeling aspirin‐d3 and mass spectrometry analysis, aspirin directly acetylates 10 HDAC proteins, including SIRT1, a well‐studied NAD+‐dependent deacetylase. Aspirin acetylates lysine 408 of SIRT1, inhibiting its deacetylase activity and impairing substrate binding. This inhibition promotes p53‐dependent apoptosis and, in knock‐in mice with the acetylation‐mimic mutant of SIRT1, helps maintain intestinal immune homeostasis by reducing pro‐inflammatory cytokine production.

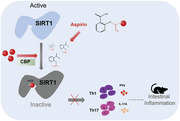

## 1 Introduction

SIRT1 is the mammalian orthologue of yeast Sir2 and has emerged as an essential regulator of aging.^[^ ^1^ ^]^ SIRT1 deacetylates diverse substrates, including PGC‐1α,^[^ ^2^ ^]^ p53,^[^ ^3^ ^]^ forkhead transcription factor (FOXO),^[^ ^4^ ^]^ NF‐κB,^[^ ^5^ ^]^ Ku70,^[^ ^6^ ^]^ and histones.^[^ ^7^ ^]^ Thus it influences gene silencing, apoptosis, stress resistance, senescence, and fat and glucose metabolism.

SIRT1 emerges as a promising biomarker for relapses and a viable therapeutic target across a range of autoimmune diseases (ADs), including inflammatory bowel disease (IBD). Its role as either an activator or inhibitor does not diminish its potential for therapeutic interventions.^[^ ^8^ ^]^ expression and activity of a multitude of transcription factors and genes. Consequently, this modulation significantly shapes the activation, differentiation, and functioning of immune cells. SIRT1 plays a major role in immunity, is required for CD4^+^ T cell tolerance and suppresses T cell activation in mice.^[^ ^9^ ^]^ SIRT1 activity can be regulated through post‐translational modification, such as phosphorylation,^[^ ^10^ ^]^ methylation,^[^ ^11^ ^]^ ubiquitination,^[^ ^12^ ^]^ S‐nitrosylation,^[^ ^13^ ^]^ and sumoylation.^[^ ^14^ ^]^ Interestingly, a recent study revealed that SIRT1 was acetylated by mass spectrometry analysis, which restored white adipose tissue in SIRT7 knockout adipocytes.^[^ ^15^ ^]^ However, it is still unknown how the acetyl group is added to SIRT1. Here we provide a potential mechanism for the functional site K408 of SIRT1 to be acetylated by aspirin or CREB binding protein (CBP) acetyltransferase. Aspirin (acetylsalicylic acid or ASA) is one of the most commonly used drugs. It is well known that aspirin can prevent pain, inflammation, and fever.^[^ ^16^ ^]^ Several studies have shown that aspirin can acetylate many proteins, including cyclooxygenase,^[^ ^17^ ^]^ serum albumin,^[^ ^18^ ^]^ fibrinogen,^[^ ^19^ ^]^ p53,^[^ ^20^ ^]^ and glucose‐6‐phosphate dehydrogenase,^[^ ^21^ ^]^ cGAS.^[^ ^22^ ^]^ Here we found that SIRT1 could be directly acetylated by aspirin or CBP. More importantly, this acetylation K408 of SIRT1 decreases the production of pro‐inflammatory cytokines and maintains intestinal immune homeostasis in mice, which reflects one piece of the puzzle of aspirin as the traditional anti‐inflammatory agent.

## 2 Results

### 2.1 Aspirin Directly Acetylates the HDACs Family

**Figure 1:**
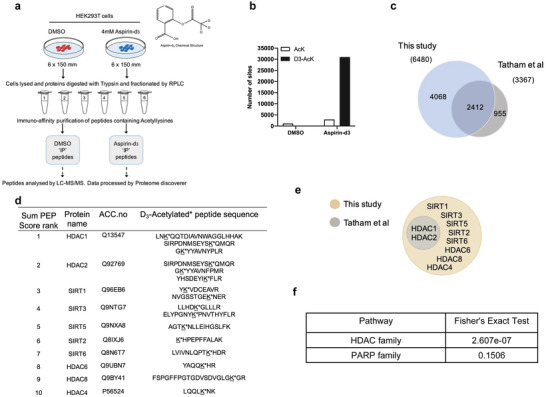
Aspirin directly acetylates the HDACs family. a) Overview of the experiment to identify protein targets of aspirin‐mediated lysine acetylation. b) Summary of numbers of AcK and d3‐AcK sites identified in peptide preparations from the two cell treatments. c) Summary of overlap between two acetylation proteomic studies: this study and (Tatham et al.) d) Ten HDAC proteins with d3‐acetylated peptide(s) qualified by MS analysis with representative acetylated peptide are shown. Perspective acetylated lysine residues within the identified peptides are marked by asterisks. e) The relationship of acetylated HDAC proteins identified in this study and (Tatham et al.) f) Preferential acetylation of enzymes in the HDAC family. Fisher's exact test of comparing acetylated proteins to total proteins shows that acetylation is much more prevalent in the HDAC family.

Aspirin (acetylsalicylate) is able to directly acetylate the side‐chain of lysine in selective protein, which may result in some unexplained drug effects. To investigate aspirin‐mediated lysine acetylation in cells, we used isotopically labeled aspirin‐d^3^ to treat HEK293T cells and followed with acetylated‐lysine peptides purification for proteomics assay by LC‐MS/MS (**Figure** **1**a). From three independent experiments, we identified 31002 acetylated d^3^‐peptides, which matched to 6480 distinct human proteins (Table S1, Supporting Information). Only 6 acetylated d^3^‐peptides were found in the DMSO treatment (Figure 1b), suggesting an actual false‐positive rate in the order of 0.5%. Compared with a recent proteomics study,^[^ ^23^ ^]^ we found 4068 unreported proteins acetylated by aspirin (Figure 1c). Tatham et al. identified 12069 aspirin‐acetylated sites in 3367 proteins from HeLa cells, and 2412 (71.6%) of these were also identified in our data set (Figure 1c), indicating that our proteomic analysis reached a high degree of coverage. Since we identified over 31000 acetylated d^3^‐peptides and is 2.57 fold of Tatham et al.’s report, which allows us to hypothesize aspirin might directly acetylate and inhibit the histone deacetylases family. Strikingly, we discovered that among 18 HDAC members, 10 HDACs were directly acetylated by aspirin (Figure 1d; Figure S1a–j, Supporting Information), and Tatham et al. only reported 2 HDACs (Figure 1e). We only identified 3 PARP members in total 17 mammalian members of the PARP family, indicating HDACs family was preferentially acetylated by aspirin compared with the total HEK293T proteome (Figure 1f). These results suggest a potential importance of HDACs in mediating known effects of aspirin on inflammation and chemoprevention. To test this possibility, we focused on SIRT1, the most conserved NAD^+^‐dependent deacetylase that is critical in the regulation of inflammation and stress resistance. Therefore, we investigated the effect of aspirin‐mediated acetylation on SIRT1.

### 2.2 Aspirin Acetylates the K408 of SIRT1 and Inhibits SIRT1 Activity

**Figure 2:**
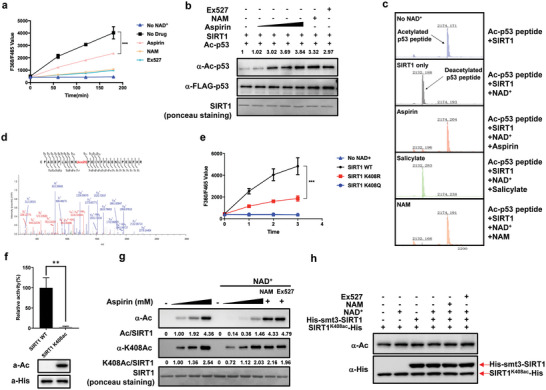
Aspirin acetylates the K408 of SIRT1 and inhibits SIRT1 activity. a) Effects of 2 mm aspirin and 1 mm NAM, 10um EX527 on SIRT1 activity. The deacetylase activities of recombinant human SIRT1 were measured by monitoring the fluorescence intensity (excitation at 360 nm and emission at 460 nm) using a substrate peptide with one end coupled to a fluorophore and the other end to a quencher. A reaction without NAD^+^ was performed as a negative control. Data are presented as mean ± s.d., n  =  3 wells, from three independent experiments. b) Representative immunoblot of three independent experiments measuring SIRT1 deacetylase activities. Acetylated p53 was incubated with indicated doses of aspirin for 3 h, then p53 acetylation was assessed by immunoblotting with anti‐p53 K382Ac antibody. c) The effects of SIRT1 deacetylation on native acetylated p53 peptide in the assay as indicated were determined by mass spectrometry. Data are representative of three independent experiments. d) D3‐acetylated SIRT1 K408 was identified by a tandem mass spectrometry. HEK293T cells were transfected with FLAG‐SIRT1 plasmids. 24 h after transfection, cells were treated with 2 mm aspirin‐D3. Then the FLAG‐SIRT1 was immunoprecipitated and subject to analysis by a MALDI‐TOF/TOF mass spectrometry. e) WT and mutants of SIRT1 were assayed for SIRT1 steady‐state kinetics. Deacetylase activity of recombinant WT SIRT1, K408R mutant, and K408Q mutant were examined and normalized against protein level. Data are presented as mean ± s.d., *n* = 3 wells, from three independent experiments. f) Deacetylase activity of recombinant WT SIRT1 and site‐specific acetylated SIRT1 was examined and normalized against the protein level; *n* = 3 biologically independent samples per group, represented as the mean s.e.m.; ^**^P = 0.0025, two‐tailed Student's *t*‐test. g) Representative immunoblot for in vitro aspirin acetylating SIRT1 with or without NAD+. Recombinant human SIRT1 was incubated with indicated doses of aspirin for 3 h with or without the presence of NAD+, then SIRT1 acetylation was assessed by immunoblotting with anti‐Pan‐acetyl‐lysine and anti‐SIRT1‐K408Ac antibody. h) Representative immunoblot of three independent experiments showing acetylated K408‐SIRT1. In vitro deacetylation assay of SIRT1 K408ac by recombinant SIRT1 purified from *E. coli* were performed and inhibitors(NAM: 500 µm, Ex527: 10 µm) of SIRT1 were added as a control. The purified precipitants were analyzed with anti‐pan‐acetyllysine and anti‐His antibody.

We first assessed the possible impact of aspirin on the activity of SIRT1 with several in vitro assays. In a fluorescence‐based deacetylation assay,^[^ ^24^ ^]^ aspirin significantly inhibited the deacetylation activity of recombinant SIRT1 (**Figure** **2**a). A general sirtuin inhibitor, NAM and a specific inhibitor of SIRT1, EX527,^[^ ^25^ ^]^ were used as positive controls. As an additional control, salicylate at 4 mm concentration could not inhibit SIRT1 deacetylase activity (Figure S2a, Supporting Information). To determine whether aspirin could inhibit the deacetylation activity of SIRT1 in the presence of a native substrate, we used acetylated p53 protein, which is generated through the overexpression of FLAG‐p53 in HEK293 cells and pulled down by incubation with p300, a known acetyltransferase of p53. Aspirin again prevented SIRT1 from deacetylating acetylated p53 protein in a dose‐dependent manner (Figure 2b), but not under salicylate treatments (Figure S2b, Supporting Information). Using an acetylated p53 peptide as a substrate in a mass spectrometry assay, we further found that aspirin, but not salicylate completely prevent SIRT1 from deacetylating the acetyl‐p53 peptide, just like NAM (Figure 2c). Moreover, in a dot blot assay using a native p53 peptide containing acetyl‐K382, aspirin but not salicylate inhibited SIRT1‐mediated deacetylation in a dose‐dependent manner (Figure S2c,d, Supporting Information). These results collectively demonstrated that aspirin directly inhibits SIRT1 deacetylase activity in vitro.

Aspirin has been shown to irreversibly inhibit cyclooxygenases' activity through the acetylation of the active site serine 530.^[^ ^17b^ ^]^ Therefore, we suspected that aspirin might inhibit SIRT1 by acetylating the active site of SIRT1. We performed mass spectrometry (LC‐MS/MS) analysis after treating HEK293T cells with isotopically labeled aspirin‐d_3_ and immunoprecipitated FLAG‐SIRT1. Strikingly, only one lysine 408 was identified as subject to acetylation by MS analysis (Figure 2d), not the two d3‐acetylated sites of SIRT1 in Figure 1c. After the alignment of different HDACs obtained from multiple species, we found that only lysine 408 is highly conserved in SIRT1 (Figure S2e, Supporting Information), not two other d3‐acetylated sites of SIRT1 in Figure 1c, implying the potentially important role of K408 in SIRT1 deacetylase activity. To evaluate the role of K408 modification in SIRT1 enzymatic activity, the acetylated residue lysine 408 was converted into arginine (to mimic deacetylation) or glutamine (to mimic acetylation). The SIRT1 K408R mutant exhibited a decreased activity, while the K408Q mutant exhibited no activity (Figure 2e). To definitively demonstrate the effect of K408 acetylation on SIRT1 activity, we employed a genetic encoding system with Nε‐acetyl‐lysine to prepare recombinant proteins in *E. coli*. This expression system produced SIRT1 proteins with 100% acetylation at K408 because of the suppression of the K408‐TAG stop codon by the Nε‐acetyl‐lysine‐conjugated amber suppressor rRNA.^[^ ^26^ ^]^ Similar to the acetyl‐mimetic SIRT1 generated by the mutation of K to Q, SIRT1‐K408‐Ac exhibited no enzymatic activity (Figure 2f). We further demonstrated that K408 was a primary function site of SIRT1 by performing an activity analysis of the K408R mutant under the aspirin treatment. We found that the activity of K408R SIRT1 is not sensitive to aspirin (Figure S2f, Supporting Information), suggesting that acetylation of K408 is responsible for aspirin‐mediated suppression of SIRT1. These data suggest that K408 is a critical site for the deacetylase activity of SIRT1 and acetylation of K408 in SIRT1 could abolish its deacetylation function.

To confirm that lysine 408 is acetylated by aspirin in cells, we generated the rabbit monoclonal antibody to recognize the acetylated K408. Western blotting analysis characterizes the specific acetylated K408 (Figure S2g, Supporting Information) and shows aspirin acetylates K408 of SIRT1 in a dose‐dependent manner (Figure 2g). To examine aspirin's effects on SIRT1 acetylation and inhibition in intact cells, H1299 cells were transfected with FLAG‐SIRT1 plasmids, and after 24 h transfection cells were treated with 0.5 mm NAM plus dose‐increased aspirin. We observed that aspirin significantly increased the acetylation of SIRT1 (Figure S2h, Supporting Information), which dramatically decreased the deacetylation activity of SIRT1 (Figure S2i, Supporting Information). Collectively, these results demonstrate that aspirin‐mediated acetylation at lysine 408 inhibits SIRT1 activity.

Since SIRT1 is a most‐investigated NAD^+^‐dependent deacetylase.^[^ ^27^ ^]^ To investigate whether SIRT1 might deacetylate itself, we incubated recombinant SIRT1 with aspirin with/without NAD^+^ supplements in vitro (Figure 2g). Using a pan‐acetyllysine antibody, we observed that aspirin could acetylate SIRT1 in a dose‐dependent manner. Interestingly, the acetylation of SIRT1 was significantly reduced in the presence of 1 mm NAD^+^ supplementation, which indicated that active SIRT1 could deacetylate acetylated SIRT1 in the presence of NAD^+^. To determine whether active SIRT1 might deacetylate acetylated K408 in SIRT1, recombinant SIRT1‐K408‐Ac was incubated with active SIRT1 and EX527 (a specific inhibitor of SIRT1) or NAM (a general inhibitor of sirtuins) in vitro. Surprisingly, active SIRT1 could not deacetylate SIRT1‐K408‐Ac (Figure 2h). Also, the purified SIRT1‐HA from HEK293 could not deacetylate the SIRT1‐K408‐Ac (Figure S2j, Supporting Information). These results implied that the aspirin‐mediated acetylation of K408 in SIRT1 could not be deacetylated by active SIRT1, which resulted in the inhibition of its deacetylation activity. We didn't rule out that other cellular deacetylases have the potential to deacetylate acK408 in SIRT1.

### 2.3 Inactivation of Acetylated K408 of SIRT1 due to Impaired Substrates Binding Affinity

**Figure 3:**
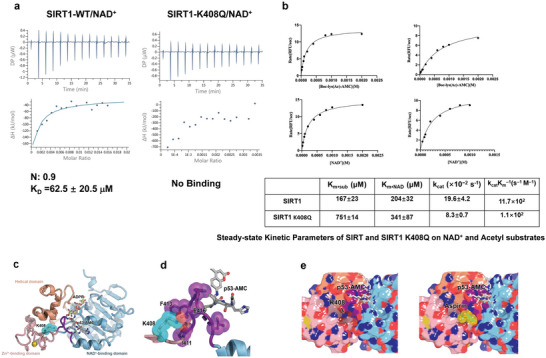
Inactivation of acetylated K408 of SIRT1 due to impaired substrates binding affinity. a) ITC measurements of binding affinities of NAD+ to the recombinant WT SIRT1 and K408Q mutant. b) Titration curves for determination of Km values of recombinant WT SIRT1 and K408Q mutant toward the indicated fluorogenic substrates and NAD+. A summary of measured Michaelis–Menten kinetic parameters for the indicated fluorogenic substrates and NAD+. c,d) Overview of the SIRT1 structure highlighting the position of K408 (in cyan‐colored spheres). The model is adapted from crystal structures of SIRT1 bound with either ADPR (PDB: 4KXQ) or a peptide substrate p53‐AMC (PDB: 5BTR). The structured loop connecting the Zn^2+^‐binding domain and the NAD‐binding domain is shown in purple. e) A close‐up view of the interactions between K408 and the connecting loop.

To elucidate the potential mechanism behind the inhibition of SIRT1 by aspirin‐mediated acetylation, an investigation into the binding affinity of recombinant SIRT1 and its K408Q mutant to NAD^+^ was conducted. The results, depicted in **Figure** **3**a, were obtained through isothermal titration calorimetry (ITC), which revealed a dissociation constant (KD) of 62.5 µM between recombinant SIRT1 and NAD^+^. Interestingly, no discernible heat absorption or release was observed during the titration of recombinant SIRT1 K408Q mutant with NAD^+^. This finding suggests that the acetylation mimic‐substitution of lysine 408 with glutamine (K408Q) significantly disrupted the binding interaction between recombinant SIRT1 and NAD^+^, thereby explaining the lack of activity exhibited by the recombinant SIRT1 K408Q mutant. Furthermore, the impact of the K408Q mutant on the fluorescent substrate was evaluated using the SIRT1 activity assay. The kinetic parameters of recombinant SIRT1 and SIRT1 K408Q mutant were determined for NAD^+^ and the fluorogenic substrate Boc‐Lys(Ac)‐AMC. Notably, the K408Q mutant led to a significant increase in the Km value for both NAD^+^ and Boc‐Lys(Ac)‐AMC (Figure 3b). To validate these findings in light of structural observations, molecular dynamics simulations of the acetylation of lysine 408 in human SIRT1 were performed using available protein structure data (PDB IDs 4KXQ^[^ ^27^ ^]^ and 5BTR^[^ ^28^ ^]^). Molecular docking analysis indicated that lysine 408 was situated within the interior of SIRT1 (Figure 3c–e), close to the p53AMC substrate‐binding domain. This positioning suggests the potential for aspirin‐mediated acetylation to inhibit the deacetylation activity of SIRT1 by affecting the critical lysine residue. Furthermore, a protein thermal shift assay revealed that the K408Q mutant of SIRT1 exhibited remarkable instability compared to wild‐type SIRT1 (Figure S3a, Supporting Information). Additionally, the K408 residue was found to be highly conserved across different histone deacetylases (HDACs) (Figure S3b, Supporting Information). Deacetylase activity assays of the K408Q mutant were performed on SIRT2 and SIRT3, resulting in no detectable activity (Figure S3c, Supporting Information). These findings suggest that aspirin has the potential to acetylate the internal lysine residues of SIRT1/2/3 and consequently impact their functional capabilities.

### 2.4 Acetylation of SIRT1 at K408 is Regulated by CBP and HDAC1

**Figure 4:**
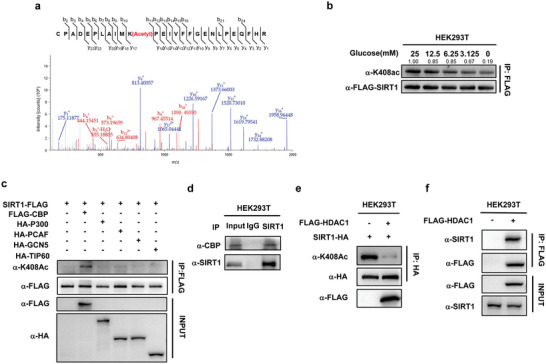
Acetylation of SIRT1 at K408 is Regulated by CBP and HDAC1. a) acetylated SIRT1 K408 was identified by a tandem mass spectrometry. HEK293T cells were transfected with FLAG‐SIRT1 plasmids without aspirin supplements. Then the FLAG‐SIRT1 was immunoprecipitated and subject to analysis by a MALDI‐TOF/TOF mass spectrometry. b) Representative immunoblotting of three independent experiments shows abundant glucose increases the SIRT1 K408ac level. The HEK293T cells were transfected with SIRT1‐FLAG, and the cell culture medium was changed to DMEM with glucose of the indicated concentration 12 h before harvest. Immunoprecipitations were performed with anti‐FLAG M2 magnetic beads. The anti‐FLAG precipitants were analyzed with anti‐SIRT1‐K408Ac antibody and anti‐FLAG. c) CBP acetylates SIRT1 in the K408 site. FLAG‐SIRT1 was coexpressed with different acetyltransferases in HEK293T cells and purified by Flag beads. d) Co‐immunoprecipitation assay detecting CBP‐SIRT1 binding in HEK293T cells. e) HDAC1 deacetylates SIRT1 in the K408 site. SIRT1‐HA was coexpressed with FLAG‐HDAC1 in HEK293T cells and purified by HA beads. f) Co‐immunoprecipitation assay detecting HDAC1‐SIRT1 binding in HEK293T cells transiently transfected with Flag‐tagged HDAC1.

Although we found aspirin directly acetylates k408 of SIRT1 in vitro, we did not rule out that the k408 of SIRT1 could be acetylated by acetyltransferase. Using mass spectrometry, we identified that the k408 of SIRT1 is acetylated in cells without aspirin supplement (**Figure** **4**a). Moreover, to determine whether ac‐K408 levels of SIRT1 in response to glucose alteration, HEK293T cells were treated with glucose in different concentrations. We found that ac‐K408 of SIRT1 was decreased under lower glucose concentrations in a dose‐dependent manner (Figure 4b). This result is consistent with a previous report that changes in the NAD^+^/NADH ratio within a cell, i.e., the cellular redox state, may influence SIRT1 activity.^[^ ^28^ ^]^ To identify the potential acetyltransferase that is responsible for SIRT1 k408 acetylation, we overexpressed CBP, p300, PCAF, GCN5, and Tip60 in HEK293T and found that the K408 acetylation of SIRT1 was elevated only after the ectopic expression of CBP, not the other acetyltransferases (Figure 4c). Mass spectrometry (LC‐MS/MS) analysis revealed a consistent increase in acetylation levels at K229 of SIRT2 in cells with ectopically overexpressed CBP (Figure S4a, Supporting Information). Interestingly, the incubation of purified SIRT1 with CBP replicated the earlier observation, confirming that CBP indeed acetylates SIRT1. (Figure S4b, Supporting Information). when CBP expression was inhibited using small interfering RNA (siRNA), a decrease in acetylation levels at K408 of SIRT1 was observed (Figure S4c, Supporting Information). We also detected a specific interaction between CBP and SIRT1 through the Co‐IP assays (Figure 4d). This result suggests that CBP is involved in the acetylation of SIRT1 at this specific lysine residue and highlights the reciprocal relationship between CBP expression and SIRT1 acetylation. Subsequently, we delved into identifying the deacetylase responsible for SIRT1 regulation. While previous studies suggested an association between SIRT1 and HDAC1 (PMID: 23852118), our objective was to ascertain whether HDAC1 functions as the deacetylase for SIRT1. To investigate, SIRT1 and HDAC1 were coexpressed in HEK293T cells, and the acetylation level of SIRT1 was examined. Our results demonstrated that the ectopic expression of HDAC1 resulted in a reduction of SIRT1 acetylation at the K408 site (Figure 4e). Furthermore, the interaction between HDAC1 and SIRT1 in HEK293T cells was corroborated through coimmunoprecipitation (Figure 4f). Collectively, our findings suggest that CBP and HDAC1 may collectively contribute to a reversible acetylation role for K408‐SIRT1 in cells.

### 2.5 SIRT1 is Required for Aspirin to Induce Apoptosis

**Figure 5:**
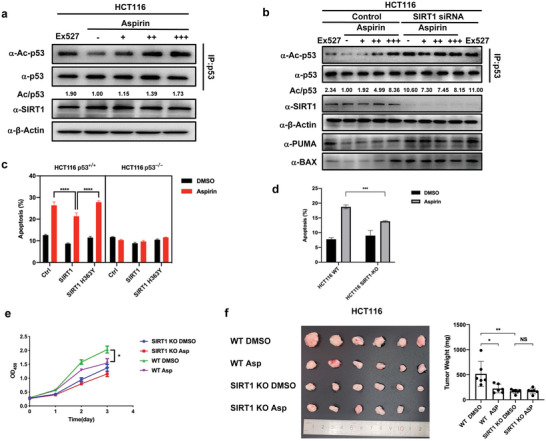
SIRT1 is required for Aspirin to induce apoptosis. a) Representative immunoblot of three independent experiments showing the analysis of SIRT1 and its substrates p53ac in HCT116 cells treated with different doses of Aspirin for 24 h. p53 acetylation was assessed after p53 immunoprecipitation. b) Representative immunoblot of three independent experiments showing the acetylated levels of downstream targets of p53. HCT116 cells were transfected with control and SIRT1 siRNAs. Cells were treated as described before and western blot analysis of SIRT1, BAX, PUMA in cell extracts, and p53 acetylation after p53 immunoprecipitation. c) HCT116(WT) and HCT116(p53‐/‐) cells were treated with DMSO or 2 mm aspirin, and apoptosis was measured by FACS. Overexpression of SIRT1 suppresses apoptosis but only in the presence of p53. Error bars represent s.d., *n* = 3. d) HCT116(WT) and HCT116(SIRT1 KO) cells were treated with DMSO or 2 mm aspirin, and apoptosis was measured by FACS. Error bars represent s.d., *n* = 3. e) Cell growth of HCT116(WT) and HCT116(SIRT1 KO) cells incubated with DMSO or 2 mm aspirin. The cell numbers were detected by the CCK8 kit. f) HCT116(WT) and HCT116(SIRT1‐KO) cells were treated with DMSO or 2 mm aspirin to perform a xenograft assay in nude mice. Tumors were photographed and weighed. Error bars represent s.d., *n* = 6.

It is well known that SIRT1 deacetylates p53 to suppress apoptosis.^[^ ^3^ ^]^ Aspirin has been reported to induce apoptosis in multiple types of cancer cells by inhibiting cyclooxygenase^[^ ^29^ ^]^ or triggering the release of cytochrome c.^[^ ^30^ ^]^ To determine whether aspirin‐mediated acetylation of SIRT1 contributes to aspirin‐induced apoptosis, we performed multiple cell‐based assays. First, we treated the colon cancer cell line HCT116 with increasing aspirin and EX527, a specific SIRT1 inhibitor. The acetylation of endogenous p53 was detected by an acetylated‐K382 p53 antibody. We found that aspirin increased p53 acetylation in a dose‐dependent manner with consistent SIRT1 protein levels (**Figure** **5**a). Similar results were observed in overexpressed p53 in HEK293T cells (Figure S5a, Supporting Information). Then, SIRT1 was knockdown in HCT116 cells or knocked out in DLD1 cells to determine whether SIRT1 is dispensable for the acetylation of p53. The acetylation of p53 did not change in SIRT1‐knockdown HCT116 cells(Figure 5b) or SIRT1‐KO DLD1 cells during aspirin treatment (Figure S5b, Supporting Information), indicating that SIRT1 is required to increase the acetylated p53 levels during aspirin treatment. Moreover, PUMA and BAX's protein levels, two other targets of p53 involved in apoptosis, were raised along with the acetylated p53 levels during aspirin treatment but not in SIRT1‐knockdown HCT116 cells (Figure 5b). We confirmed similar data of increased transcriptional levels of PUMA and BAX with aspirin treatments in a dose‐dependent manner (Figure S5c, Supporting Information). This is consistent with previous reports that acetylated p53 activates its transcription activity to induce apoptosis.^[^ ^31^ ^]^ Finally, we performed apoptosis analysis to assess whether the SIRT1‐p53 axis is involved in aspirin‐induced apoptosis. p53^+/+^ or p53^−/−^ HCT116 cells were treated with aspirin. Overexpressing SIRT1 in p53^+/+^ HCT116 decreased apoptosis levels during aspirin treatment (Figure 5c), compared with overexpressing the vector control or SIRT1‐H363Y (an enzymatically dead mutant). However, in p53^−/−^ HCT116, aspirin treatment did not alter the apoptosis levels (Figure 5c). Furthermore, apoptosis induced by aspirin in SIRT1‐KO HCT116 cells was significantly reduced compared with that in HCT116 WT cells (Figure 5d; Figure S5d, Supporting Information). The result shows no different elevation in apoptosis rates induced by the DMSO group between wild‐type and SIRT1 knockout cells, because we established the SIRT1‐KO HCT116 cell line using CRISPR/Cas9 system. A single clone was obtained and was fully validated for lack of expression and normal apoptosis levels. Cell proliferation assays also confirmed that SIRT1 is the primary target for aspirin inhibition of cell growth (Figure 5e). Subsequent to the administration of treatments using either DMSO or aspirin to WT and SIRT1 knockout (KO) HCT116 cells, a xenograft assay was conducted in nude mice (Figure 5f). The outcome indicates that aspirin treatment effectively inhibited tumor formation in WT HCT116 cells when subcutaneously implanted in nude mice. Conversely, in SIRT1 KO HCT116 cells, aspirin failed to suppress tumor growth. This outcome strongly implies that the capacity of aspirin to induce tumor cell apoptosis is contingent upon the presence of the SIRT1 protein. These results strongly indicate that the SIRT1‐p53 axis is indispensable for aspirin‐induced cell apoptosis (Figure S5e, Supporting Information).

### 2.6 Acetylated SIRT1 Maintains Intestinal Immune Homeostasis

**Figure 6:**
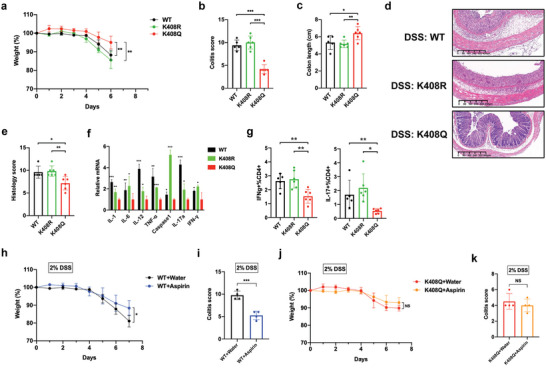
Acetylated SIRT1 maintains intestinal immune homeostasis. a) Changes of body weights in the mice of each group. *n* = 5 (WT), *n* = 6 (K408R^+/+^), and *n* = 6 (K408Q^+/−^).Error bars represent mean ± s.d. ^*^ *p* < 0.05, ^**^ *p* < 0.01, ^***^ *p* < 0.001, ns = not significant; two‐tailed, unpaired *t*‐test. b) colitis scores *n* = 5 (WT), *n* = 6 (K408R^+/+^), *n* = 6 (K408Q^+/−^).Error bars represent mean ± s.d. ^*^ *p* < 0.05, ^**^ *p* < 0.01, ^***^ *p* < 0.001, ns = not significant; two‐tailed, unpaired *t*‐test. c) colon length. *n* = 5 (WT), *n* = 6 (K408R^+/+^), *n* = 6 (K408Q^+/−^).Error bars represent mean ± s.d. ^*^ *p* < 0.05, ^**^ *p* < 0.01, ^***^ *p* < 0.001, ns = not significant; two‐tailed, unpaired *t*‐test. d) Representative H&E‐stained colonic tissue of WT,K408R^+/+^ and K408Q^+/−^ mice. e) Histological scoring of sections in (f). *n* = 5 (WT), *n* = 6 (K408R^+/+^), *n* = 6 (K408Q^+/−^).Error bars represent mean ± s.d. ^*^ *p* < 0.05, ^**^ *p* < 0.01, ^***^ *p* < 0.001, ns = not significant; two‐tailed, unpaired *t*‐test. f) The mRNA level change of IL‐1, IL‐6, IL‐12, TNF‐α,Caspase1, IL‐17a, and IFN‐γ were determined by Real‐time PCR. Data are mean ± s.d, two‐tailed, unpaired *t*‐test. g) Intestinal infiltrating lymphocyte were isolated and analyzed by Flow cytometry. The percentage of IFN‐γ^+^ or IL17^+^ T cells in CD4^+^ T cells were quantified. *n* = 5 (WT), *n* = 6 (K408R^+/+^), *n* = 6 (K408Q^+/−^). Error bars represent mean ± s.d. ^*^ *p* < 0.05, ^**^ *p* < 0.01, ^***^ *p* < 0.001, ns = not significant; two‐tailed, unpaired *t*‐test. h) Changes of body weights in the mice of each group. *n* = 4 (WT+vehicle), *n* = 4 (WT+aspirin). Error bars represent mean ± s.d. ^*^ *p* < 0.05, ^**^ *p* < 0.01, ^***^ *p* < 0.001, ns = not significant; two‐tailed, unpaired *t*‐test. i) colitis scores. *n* = 4 (WT+vehicle), *n* = 4 (WT+aspirin). Error bars represent mean ± s.d. ^*^ *p* < 0.05, ^**^ *p* < 0.01, ^***^ *p* < 0.001, ns = not significant; two‐tailed, unpaired *t*‐test. j) Changes of body weights in the mice of each group. *n* = 4 (K408Q+vehicle), *n* = 4 (K408Q+aspirin). Error bars represent mean ± s.d. ^*^ *p* < 0.05, ^**^ *p* < 0.01, ^***^ *p* < 0.001, ns = not significant; two‐tailed, unpaired *t*‐test. k) colitis scores. *n* = 4 (K408Q+vehicle), *n* = 4 (K408Q+aspirin). Error bars represent mean ± s.d. ^*^ *p* < 0.05, ^**^ *p* < 0.01, ^***^ *p* < 0.001, ns = not significant; two‐tailed, unpaired *t*‐test.

Aspirin is appreciated for its analgesic and anti‐inflammatory actions.^[^ ^32^ ^]^ Recent studies uncovered that aspirin plays a protective role in colitis‐associated colorectal cancer.^[^ ^33^ ^]^ To further investigate whether SIRT1‐K408 acetylation could influence the pathogenesis of IBD, we generated the whole‐body K408R (mimic‐deacetylation) and K408Q (mimic‐acetylation) knockin mice by CRISPR/Cas9‐mediated genome engineering. Interestingly, we failed to obtain homozygous K408Q‐KI (K408Q^+/+^) mice, and the heterozygous K408Q‐KI mice (K408Q^+/−^) showed significantly lower fertility than wild‐type mice (data not shown). WT, K408R ‐KI (K408R^+/+^) and K408Q‐KI (K408Q^+/−^) mice were treated with DSS to induce colitis. While WT and K408R^+/+^ mice showed significant weight loss, a proxy for morbidity, during DSS treatment, K408Q^+/−^ mice displayed less weight loss (**Figure** **6**a). In agreement, the disease scores (DAI) of the K408Q^+/−^ mice were significantly decreased compared with those of the WT and K408R^+/+^ group (Figure 6b). The colons of K408Q^+/−^ mice were also significantly longer than WT and K408R^+/+^ group (Figure 6c). To examine whether the disease conditions correlated with inflammation and tissue damages, we performed hematoxylin and eosin (H&E) staining on paraffin‐embedded colon tissues and found that K408Q^+/−^ mice showed a significant decrease of immune infiltration and tissue damages in the colons compared to WT and K408R^+/+^ mice (Figure 6d), which was summarized as histopathologic scores (Figure 6e). We then assessed inflammation in the colon by examining the expression of inflammatory cytokines. In line with the reduced disease severity of K408Q^+/−^ mice, we found that the expression of IL‐1, IL‐6, IL‐12, TNF‐α,Caspase1, IL‐17a and IFN‐γ was significantly lower in K408Q^+/−^ mice than in WT and K408R^+/+^ mice (Figure 6f), suggesting SIRT1‐K408 acetylation reduced intestinal inflammation. Because T_H_1 and T_H_17 cells play an important role in DSS‐induced colitis, we examined T_H_1 and T_H_17 cells in DSS‐treated mice and observed decreased number of T_H_1, and T_H_17 cells in K408Q^+/−^ mice compared with WT and K408R^+/+^ mice (Figure 6g). This suggests that the increased SIRT1 K408 acetylation in T_H_1 and T_H_17 cells damped T_H_1 and T_H_17 cell differentiation in the inflamed intestines of DSS‐treated mice, leading to decreased production of inflammatory cytokines. To gain further insight into the crucial involvement of SIRT1 K408 acetylation in aspirin's anti‐inflammatory mechanisms, we conducted a study using both wild‐type (WT) and K408Q mutant mice in a DSS‐induced colitis murine model. Our experimental approach included administering aspirin at a dose of 25 mg Kg^−1^ through their drinking water regimen. As anticipated, we administered aspirin seven days prior to the initiation of DSS treatment, resulting in a notable reduction in disease severity among WT mice. This reduction was evidenced by an increase in body weight and a corresponding decrease in colitis scores (Figure 6h,i). Intriguingly, these protective effects of aspirin were conspicuously absent in K408Q mice (Figure 6j,k). This discovery underscores the pivotal and irreplaceable role played by SIRT1 K408 acetylation in orchestrating the anti‐inflammatory potency of aspirin within the context of DSS‐induced colitis murine models. Together, these results demonstrate that Aspirin‐acetylated SIRT1 can attenuate inflammatory responses in DSS‐induced mice and maintain intestinal immune homeostasis.

## 3 Discussion

Aspirin is the most reliable anti‐inflammatory effect agent in the world. Several studies have reported the acetylation substrates of aspirin, including cyclooxygenases,^[^ ^17b^ ^]^ serum albumin,^[^ ^18^ ^]^ fibrinogen^[^ ^19^ ^]^ and cGAS.^[^ ^22^ ^]^ Here, we provide additional interesting evidence that SIRT1, an NAD^+^‐dependent deacetylase, can be acetylated by aspirin or acetyltransferase CBP. The activity of SIRT1 must be rigidly controlled in response to various cellular stimuli. Interestingly, SIRT1 plays a dose‐dependent effect in colon cancer formation^[^ ^34^ ^]^ and adipogenesis.^[^ ^15^ ^]^ SIRT1 is regarded as a critical immune modulator by suppressing inflammation through deacetylating several substrates, such as NF‐κB, STAT3, PDK1, and PTEN.^[^ ^35^ ^]^ SIRT1 has also been shown to play the immune suppression function and promote tumor progression through inhibiting glycolytic activation and preventing the differentiation of myeloid‐derived suppressor cells (MDSCs) to M1 phenotype.^[^ ^36^ ^]^ Activation of SIRT1 usually suppresses T‐cell immune responses by inhibiting the NF‐κB and AP‐1.^[^ ^37^ ^]^ Here we demonstrated that under inflammatory stress acetylated‐K408 SIRT1 suppresses the development of T_H_1 and T_H_17 cells in the gut, two key types of pro‐inflammatory T cells, and reduces the production of pro‐inflammatory cytokines. Moreover, aspirin administration plays a protective role in DSS‐induced colitis in WT mice, but not in K408Q mice. Our data underscore the significance of SIRT1‐K408 acetylation in maintaining intestinal immune homeostasis. Given that aspirin targets the conserved K408 residue of SIRT1, our study unveils a novel mechanism through which aspirin mitigates inflammation. This discovery bolsters the role of aspirin as a globally recognized therapeutic agent for its potent anti‐inflammatory effects.

SIRT1 has been demonstrated to play the opposite effect as an oncoprotein or a tumor suppressor under different conditions. Upregulated SIRT1 is also involved in cutaneous T‐cell lymphoma^[^ ^38^ ^]^ and inhibition of SIRT1 induces growth arrest and apoptosis in Hodgkin lymphoma cells.^[^ ^39^ ^]^ Therefore, SIRT1 plays a critical antiapoptotic role in lymphoma, similar to regular aspirin use in preventing colorectal cancer. The key antiapoptotic role of SIRT1 is exhibited through deacetylating the C‐terminal lysine‐382 residue in p53 in an NAD^+^‐dependent manner.^[^ ^3^ ^]^ This reaction decreases the transcriptional activity of p53 and leads to the downregulation of its downstream proteins such as PUMA and Bax. Therefore, SIRT1 may exert an inhibitory effect on p53‐dependent apoptosis. In this study, we present an interesting mechanism wherein aspirin may impede SIRT1 activity by acetylating its functionally significant interior site, thereby triggering apoptosis in tumor cells.

Beyond the scope of aspirin, our investigation has unveiled CBP as the agent responsible for K408 acetylation of SIRT1 within cellular environments. Remarkably, the inhibition of CBP/p300 has been corroborated as a means to attenuate human Th17 responses and manifest potential antitumor properties.^[^ ^40^ ^]^ It is pertinent to underscore that the plausible involvement of alternative acetyltransferases, capable of modulating K408 of SIRT1 in vivo, has not been conclusively ruled out. Intriguingly, our experimental findings have illustrated that active SIRT1 fails to effectuate deacetylation of acetylated‐K408 SIRT1 in vitro. Furthermore, we have yet to exclude the possibility of other deacetylases with the potential to mediate the deacetylation of acetylated‐K408 SIRT1. The imperative investigation of the intricate regulatory mechanisms governing acetyltransferases and deacetylases in the K408‐SIRT1, particularly in response to anti‐inflammatory functions, emerges as a compelling avenue for future research.

We have elucidated that the K408 site resides within the interior of SIRT1 and plays a pivotal role in its deacetylase activity. This site is susceptible to direct acetylation by aspirin or CBP. Remarkably, acetylation at K408 remains resistant to deacetylation by SIRT1. Furthermore, our investigation revealed a high degree of conservation of the K408 site across various HDACs. Notably, the K408Q mutant (mimicking acetylated status) in SIRT2 and SIRT3 displayed a loss of deacetylase activity (Figure S3b,c, Supporting Information). These findings suggest that aspirin or CBP might directly acetylate members of the HDAC family, thereby inhibiting their deacetylase functions, underscoring their crucial role in vivo. Our discoveries provide novel insights into the role of aspirin/CBP‐mediated acetylation of SIRT1 in maintaining intestinal immune homeostasis.

## 4 Experimental Section

### Proteomic Procedures

HEK293T cells were cultured in DMEM medium supplemented with 10% fetal bovine serum (BI), 100 units/mL penicillin, and 100 mg mL^−1^ streptomycin (Sangon Biotech). In order to accumulate d3‐acetylated proteins, cells were treated with 4 mm d3‐aspirin dissolved in DMSO for 6 h. Six fully confluent 150‐mm dishes of cells were collected for each experiment. The experiments were repeated from three individual samples.

### Protein Reduction, Alkylation, and FASP Digestion

Cells were washed three times with ice‐cold PBS and then re‐suspended with 8 m guanidine hydrochloride and 100 mm Tris‐HCl, pH 8.0, incubated at 95 °C for 5 min, and further ultra‐sonicated. The homogenate was centrifuged at 20 000 g for 20 min, and then the supernatant was determined with Bradford assay. Proteins were reduced, alkylated, and digested with trypsin using the FASP digestion.^[^ ^41^ ^]^

### Off‐Line Reverse‐Phase HPLC Fractionation

Peptides were fractionated on a Gemini‐NX C18 column (3 µm, 110 Å, 4.6 mm i.d. × 250 mm (Phenomenex, USA)) using Dionex UltiMate‐3000 HPLC system (Thermo Fisher Scientific, USA). 6 mg of peptides were dissolved with 10 mm NH_4_HCO_3_ and fractionated using a 40 min gradient. Eluted peptides were collected at 1‐min intervals. The eluate from the sixth minute to the 36th minute was combined into six fractions and then speed vac‐dried before following acetylation enrichment.

### Acetylated‐Lysine Peptides Enrichment

The enrichment procedure was employed as described in the vendor protocol. Briefly, the dried peptide mixtures (≈1 mg each fraction) were dissolved in 200 µL IP buffer and incubated with 10 µL of pre‐washed Anti‐acetyllysine antibody‐conjugated agarose beads (PTM‐104, PTM Biolabs, China) at 4 °C for 12 h. After four times washing with IP buffer, the beads were transferred onto a pipette with a microfiber frit. The beads were further washed three times with MilliQ by 400 g centrifuge, and then the enriched acetylated peptides were eluted for three times with 50 µL of elution buffer each, and once with 50 µL of 10% ACN. The eluates were pooled and speed vac‐dried before MS analysis.

### LC‐MS/MS Analysis

LC‐MS/MS analysis was performed using a nanoflow EASY‐nLC 1200 system coupled to an Orbitrap Explroris 480 mass spectrometer with FAIMS Pro device (Thermo Fisher Scientific, USA). Samples were first loaded and analyzed on a homemade analytical column (75 µm i.d. × ≈20 cm; 1.9 µm, ReproSil‐Pur 120 C18‐AQ, Dr. Maisch GmbH, Germany) packed using the Flashpacking approach.^[^ ^42^ ^]^ The mobile phases consisted of solvent A (0.1% formic acid) and solvent B (0.1% formic acid in 80% ACN). The peptides were eluted using the following gradients: 5–44% B in 43 min, 44–60% B in 5 min, 60–100% B in 2 min, and 100% B for 10 min at a flow rate of 200 nL mi^−1^n. Data acquisition mode was set to obtain one MS scan at a resolution of 120 000 (m/z 350–1600) and followed by MS/MS scans using HCD (cycle time of 3s; normalized collision energy (NCE) of 30; isolation width of 0.7 m/z; resolution of 7500; maximum injection time of 22 ms). Two FAIMS experiments of −45 CV and −65 CV were employed, and data were acquired using a top speed of 1 sec/CV for each FAIMS experiment. Dynamic exclusion of 30 s was applied with a precursor mass tolerance of 10 ppm.

### MS/MS Data Analysis

The raw data were analyzed by Proteome Discoverer (version 2.4, Thermo Fisher Scientific) using Sequest HT. The human SwissProt database (202003, 20365 sequences) was downloaded from UniProt. The processing and consensus workflow was set up according to the incorporated analysis templates. The parameters applied in the processing workflow were: trypsin/P as the enzyme; up to four missed cleavage sites were allowed; 10 ppm mass tolerance for MS and 0.05 Da for MS/MS fragment ions; propionamidation on cysteine as fixed modification; oxidation on methionine, acetylation and d3‐acetylation on lysine, oxidation on methionine, acetylation on protein N‐Terminal, Met‐loss on protein N‐Terminal, Met‐loss+Acetyl on protein N‐Terminal as variable modification. The incorporated Percolator in Proteome Discoverer was used to validate the search results and only the hits with FDR ≤ 0.01 were accepted for further analyses.

### Quantitative Polymerase Chain Reaction (qPCR)

Cells were harvested and the total RNA was extracted with TRIzol (Thermo Fisher Scientific) according to the manufacturer's protocol. The diluted RNA was reverse‐transcripted (ReverTra Ace qPCR RT Master Mix with gDNA Remover, FSQ‐301, TOYOBO) and analyzed with qPCR (SYBR Green Realtime PCR Master Mix, QPK‐201) for the expression of genes on CFX Connect Real‐time PCR Detection System (BIO‐RAD). The primers were synthesized from GenePharma and the primers are shown in Table S2 (Supporting Information).

### Generation of Rabbit Monoclonal Antibody of SIRT1 K408Ac

A monoclonal antibody specific to SIRT1 K408Ac was prepared commercially from immunizing rabbits at Abcam Shanghai Company (antigen peptide: DEPLAIMK(acetyl)PEI, 1:1000).

### Protein Expression and Purification

DNA fragments of *Homo sapiens* SIRT1 or SIRT3 were amplified by PCR and cloned into the vector pET28a. The mutants were generated by KOD‐Plus‐Mutagenesis Kit (TOYOBO) and confirmed by DNA sequencing. For SIRT1, Residues 183–510 and residues 641–665 were reserved and connected with a linker (GGGSGGGS). For SIRT3, Residues 118–395 were cloned. The protein was expressed in *Escherichia coli* BL21 (DE3) cells as an N‐terminal fusion to a hexahistidine affinity tag and a SUMO protein tag. A single colony was inoculated in LB media containing 100 µg mL^−1^ kanamycin at 37 °C, 220 r.p.m. overnight. Then transfer the cells into 2 liters of LB media supplemented with kanamycin. Protein expression was induced at an OD_600_ of ≈0.6 with 500 µm IPTG for 16–18 h at 18 °C. Cells were collected by centrifugation, and the pellet was resuspended in Binding Buffer (50 mm Tris‐HCl, pH 7.4, 500 mm NaCl, 20 mm Imidazole). After the rupture by High‐Pressure Homogenizer, the lysate was centrifuged for 1 h at 18000 r.p.m. The cleared lysate was loaded onto a HisTrap column (GE Healthcare) that equilibrated with Binding Buffer and attached to an ÄKTA FPLC system (GE Healthcare). The column was washed with five column volumes of the binding buffer and eluted with elution buffer (50 mm Tris‐HCl, pH7.4, 500 mm NaCl, 500 mm Imidazole). The eluted protein was digested with Ulp1 protease to remove the N‐terminal His tag and SUMO protein tag at 4 °C, 1 h. The digested protein was diluted with binding buffer, and then loaded on a second HisTrap Column equilibrated with Binding Buffer. The untagged protein was eluted by the binding buffer and concentrated to 15–20 mg mL^−1^ in the dialyzing buffer (10 mm Tris‐HCl, pH 7.4, 100 mm NaCl, 5 mm β‐mercaptoethanol).

### Purification of Site‐Specific Acetylated SIRT1 Recombinant Proteins

The site‐specific acetylated SIRT1 recombinant proteins were generated according to Neumann et al. Wild‐type SIRT1 was cloned into pTEV‐8 (pET‐21b backboned with tobacco etch virus cleavage site), producing a C‐terminal His_6_‐tagged construct. By incorporating an amber codon at Lys‐408 (AAG to TAG by site‐directed mutagenesis), pTEV‐SIRT1 encoded for a homogeneous pool SIRT1Ac, was produced. The three plasmids (pTEV‐8 with the expressed gene, pCDF pylT‐1, and pAcKRS) were transformed into *Escherichia coli* BL21 (DE3) cells. A single colony was inoculated in LB media containing 50 µg mL^−1^ spectinomycin, 50 µg mL^−1^ kanamycin, and 150 µg mL^−1^ ampicillin at 37 °C, 220 r.p.m. overnight. Overnight cultures were subcultured 1:100 into 2 liters of 2x YT media supplemented with spectinomycin, kanamycin, and ampicillin. Cultures were grown at 37 °C with shaking to an OD_600_ of ≈0.6, 20 mm nicotinamide (NAM) and 10 mm N^ε^‐acetyllysine (Sigma‐Aldrich) were added and 30 min later, the protein expression was induced at 18 °C overnight by adding 0.5 mm IPTG. Cells were harvested by centrifugation after induction and were washed with ice‐cold PBS containing 20 mm nicotinamide. Then the pellet was resuspended in the buffer (50 mm Tris‐HCl, pH 7.4, 500 mm NaCl, 20 mm Imidazole, 20 mm nicotinamide) containing DNase I (25 µg mL^−1^) and PMSF (0.5 mm). After the rupture by a high‐pressure homogenizer, the lysate was centrifuged for 1 h at 18000 rpm. The cleared lysate was loaded onto a HisTrap column (GE Healthcare) that was equilibrated with binding buffer and attached to an ÄKTA FPLC system (GE Healthcare). The column was washed with five column volumes of the binding buffer and eluted with elution buffer. The eluted protein was concentrated in the dialyzing buffer.

### Preparation of Native Full‐Length Ac‐Lys382‐p53

The full‐length p53 cDNA was cloned into the pCDNA3.0 with a FLAG tag at the C terminus. pCDNA3.0‐p53‐FLAG was co‐transfected with p300 into H1299 cells, and 5 mm nicotinamide and 10 µm MG132 was supplemented during the cell culture. Cells were lysed in 0.5% Nonidet P40 buffer containing 50 mm Tris‐HCl (pH 7.5), 150 mm NaCl, and multiple protease inhibitors (PMSF 1 mm, Aprotinin 1 µg mL^−1^, Leupeptin 1 µg mL^−1^, Pepstatin 1 µg mL^−1^, Na_3_VO_4_ 1 mm, NaF 1 mm), TSA (2.5 mm) and NAM (5 mm). Cell lysates were incubated for 3 h at 4 °C with anti‐FLAG M2 agarose (Sigma A2220) after removing debris by centrifuging at 4 °C, 13 000 r.p.m. for 15 min. The beads were washed three times with lysis buffer and eluted with 3X FLAG peptide according to the manufacturer's protocol.

### SIRT1 Deacetylase Activity Assay

For determination of the relative SIRT1 deacetylase activity in vitro, purified SIRT1 protein was incubated with 200 µm Boc‐Lys(Ac)‐AMC (Enzo) under the following standard reaction condition: 50 mm Tris‐HCl, pH 9.0, 4 mm MgCl2, 0.2 mm DTT, 1 mm NAD+, and 1% DMSO. Reactions were carried out at 37 °C for the indicated time. For determination of enzyme kinetic parameters, the reaction contained a saturating concentration of NAD+ (1 mm) while varying Boc‐Lys(Ac)‐AMC concentrations (0–2 mm). The reactions were incubated for 60 min at 37 °C. Then 50 µl trypsin solution (0.5 mg mL^−1^) was added and the reaction mixture was incubated at 37 °C for 1 h. The deacetylation‐dependent fluorescent signal was determined using a 360‐nm excitation laser and a 460‐nm emission filter on a fluorescence plate reader. Background control reactions were performed in the absence of enzyme. All of the reactions were performed in triplicate. Km and kcat values were obtained by fitting the data to the Michaelis–Menten equation.

### SIRT1 Deacetylase Assay with Full‐Length Ac‐Lys382‐p53 Monitored by Western Blot analysis

The SIRT1 assay mixture (45 µl) containing 50 mm Tris‐HCl, pH 9.0, 4 mm MgCl_2_, 0.2 mm DTT, 1 mm NAD^+^, 1% DMSO, and 0.5 µg SIRT1 was mixed with 5 µl eluted full‐length Ac‐Lys382‐p53, and the reaction was carried out at 37 °C for 3 h. Western blot analyses were conducted after the reaction. Ac‐Lys382‐p53 and total p53 were detected by using a 1:1000 rabbit anti‐acetyl‐Lys382‐p53 antibody (Abcam) and a 1:1000 rabbit anti‐p53 antibody (Proteintech). The secondary antibody used was 1:5000 IPKine HRP, Mouse Anti‐Rabbit IgG LCS (Abbkine).

### SIRT1 Deacetylase Assay with Native Ac‐p53 Peptide Monitored by MS and Dot Plot Analysis

Native Ac‐p53 peptide (Ac‐Lys‐Lys‐Gly‐Gln‐Ser‐Thr‐Ser‐Arg‐His‐Lys‐(Ac‐Lys)‐Leu‐Met‐Phe‐Lys‐Thr‐Glu‐Gly‐NH_2_) was synthesized from GL Biochem. The SIRT1 reaction (50 µl) containing 50 mm Tris‐acetate, pH7.4, 150 mm NaCl, 1 mm NAD^+^, 1 mm DTT, 0.01% Triton X‐100, 1 µg SIRT1, and 50 µm native Ac‐p53 peptide was carried out at 37 °C for 3 h. The reaction was quenched with trifluoroacetic acid to a final concentration of 2%, centrifuged at 16 000 X g for 10 min, and detected Ac‐p53 peptide and deacetylated‐p53 peptide by MALDI‐TOF/TOF Mass Spectrometry. The dot plot was conducted by spotting 2 µl of sample onto a nitrocellulose membrane (GE Healthcare). Ac‐Lys382‐p53 was detected as same as Western blot analysis.

### SIRT1 Acetylation Assay

The acetylation assay was carried out with 50 mm Tris‐HCl, pH 9.0, 4 mm MgCl_2_, 0.2 mm DTT, 1% DMSO, 1 µg SIRT1, and Aspirin at an indicated concentration or 0.1 mm acetyl‐CoA at 37 °C, 3 h. The acetylated SIRT1 was detected by Western blot by using an Acetylated‐Lysine antibody (Cell Signaling Technology) and total SIRT1 was detected by ponceau staining.

### SIRT1 Acetylation Detected by MS

HCT116 or HEK293T cells were transfected with FLAG‐SIRT1 and treated with 4 mm Aspirin or 2 mm Aspirin‐D3 for 24 h and then the FLAG‐SIRT1 proteins were immunoprecipitated. The proteins were resolved by electrophoresis and the gel slices that contain the desired bands were collected and analyzed by MALDI‐TOF/TOF Mass spectrometry.

### ITC Measurements

All ITC measurements were carried out at 25 °C using a PEAQ‐ITC (MicroCal LLC). A total of 18 injections of 20 um protein, with a 0.4‐µL first drop (data not used) and 2‐µL subsequent drops, was titrated into a 200‐µL well of 0.2 mm NAD solution. The reference power was set at 10 µCal/sec, the initial delay was set at 60 sec, and the sample cell was stirred at 750 rpm. Injections were carried out at 0.5 µL sec^−1^ with an interval of 150 sec between injections. The titrant and the protein well solution were in the same buffer (20 mm HEPES at pH 7.5, 150 mm NaCl, 10% glycerol). Buffer control for each experiment was performed under the same conditions without protein in the titrant, and the measured background heat was subtracted from the integrated data. All of the data were integrated and analyzed using a one‐site model with the MicroCal PEAQ‐ITC analysis software

### The PI and Annexin V Staining for Cell Apoptosis Detection

HCT116 cells were transfected with HA‐SIRT1, HA‐SIRT1 H368Y mutant or control plasmid, respectively. After treatment with 2 mm Aspirin for 24 h, the cells were harvested, stained with propidium iodide and anti‐annexin‐V antibody, and analyzed by FACS.

### Cell Proliferation

HCT116 cells were seeded in 96‐well plates at the density of 1000 cells per well. 12 h after seeding, varying concentrations of Aspirin were added to the culture medium. The cells were then cultured for another 24, 48, 72, or 96 h. The supernatant was removed, and 100 µl medium containing 10 µl of CCK8 was added to each well for incubating another 3 h at 37 °C. The culture plates were then shaken for 10 min and the optical density (OD) values were read at 450 nm.

### Xenograft Studies

HCT116 stable cell lines with SIRT1 knockout(KO) and HCT116 WT cell lines were prepared. Treatments were administered with either DMSO or aspirin to both wild‐type and SIRT1 KO HCT116 cells to perform a xenograft assay in nude mice. In all, 1 × 106 cells in PBS were subcutaneously injected into each nude mice (male, 6–7 ‐week‐old), purchased from Shanghai Biomodel Organism Science & Technology Development Co. Ltd. Three weeks later, all mice were sacrificed and tumors were dissected, photographed, and weighed. Mouse experiments were done in compliance with the Institutional Animal Care and Use Committee guidelines at Fudan University.

### Mice

All mice were housed on a 12‐h light/dark cycle, and male mice were used for the experiment. All mice were in the C57BL/6j background. For the construction of a mouse strain with the K400R and K400Q(corresponding to K408R and K408Q in human *Sirt1*) knock‐in allele in *Sirt1*, CRISPR/Cas9 technology was adopted and homologous recombination repair method was used to introduce the target point mutation. The brief process is as follows: Cas9 mRNA and guide RNA were obtained by in vitro transcription, oligo donor DNA was obtained by synthesis, and Cas9 mRNA, gRNA, and donor DNA were microinjected into fertilized eggs of C57BL/6J mice to obtain F0 generation mice.

Oligo donor DNA sequence:

K400R:GTAGTTCCTCGGTGCCCTAGGTGCCCAGCTGATGAGCCACTTGCAATCATGAGGCCAGAGATTGTCTTCTTTGGTGAAAACTTACCAGAACAGTTTCATAGAGCCATGAAGTATGACAAA
K400Q:GTAGTTCCTCGGTGCCCTAGGTGCCCAGCTGATGAGCCACTTGCAATCATGCAGCCAGAGATTGTCTTCTTTGGTGAAAACTTACCAGAACAGTTTCATAGAGCCATGAAGTATGACAAA
gRNA sequence: GACAATCTCTGGCTTCATGATGG

### Intestinal Infiltrating Lymphocyte Isolation and Analysis

Intestinal infiltrating lymphocytes were isolated as described before.^[^ ^43^ ^]^ Briefly, the colon was cut open longitudinally and into small pieces and incubated for 30 min in wash buffer (RPMI 1640, 3%FBS, 0.3% penicillin/streptomycin, 1 mm ETDA, 1 mm DTT) at 37 °C with 220r.p.m. shaking to obtain intraepithelial lymphocyte (IEL). IEL and colon tissue were collected, washed, and incubated in digestion buffer (RPMI 1640, 3%FBS, 0.3% penicillin/streptomycin, 0.1 mg mL^−1^ DNase I (A610099, Sangon Biotech) 0.5 mg mL^−1^ Collagenase IV (17104019, Gibco)) for 30 min at 37 °C with 220 r.p.m. shaking. Intestinal infiltrating lymphocytes were further isolated with 32.5% Percoll (17544501, Cytiva) by density gradient centrifugation. After single cells were activated by activation buffer containing 50 ng mL^−1^ phorbol myristate acetate (PMA, 158693, MCE), 1 µm ionomycin (100898, MCE), and 5 µg mL^−1^ brefeldin A (113499, MCE) for 4 h, cells were blocked with 0.5 µg mL^−1^ anti‐CD16/32 antibody, and then were stained for the surface marker (ZombieNIR, TCRb‐BV421, CD3e‐PE, TCRgd‐APC, CD4‐efluorescence506, CD45‐PerCP Cy5.5). Cells were then fixed and permeabilized with eBioscienceTM Foxp3 / Transcription Factor Staining Buffer Set (00‐5523‐00, Invitrogen), followed by staining with IL17A‐FITC, IFN‐γ‐PE Cy7. Flow cytometry was performed with a Gallios Flow Cytometer (Beckman Coulter) and data were analyzed with FlowJo software (10.8.1).

### Establishment and Evaluation of DSS‐Induced Colitis

Eight‐week‐old male mice with similar body weights were selected for the experiment, and Experimental colitis was induced by adding 2% DSS (36000‐50,000 Da, MP Biomedicals, Solon, USA) in drinking water for 10 days (from day 0 to day 9). Aspirin was dissolved in water and administered daily in the drinking water. A daily water consumption of 0.15 L kg^−1^ body weight was used for dose calculation. For the mouse DSS‐induced colitis models, an aspirin dose of 25 mg/kg/d was administered 7 days before DSS initiation via drinking water. The animals in the control groups were received normal drinking water.The health status of the mice was observed every day, and the disease activity index (DAI) was scored according to the degree of weight loss, stool characteristics, and degree of stool bleeding. The DAI was scored using a unified scoring standard. For weight loss, 0, 1–5, 5–10, 10–15, and >15 were scored as 0, 1, 2, 3, and 4 points, respectively. For stool traits, normal, loose, and watery stool were scored as 0, 2, and 4 points, respectively. For bloody stools, normal, occult blood‐positive, and macroscopic blood stools were scored as 0, 2, and 4 points, respectively.^[^ ^44^ ^]^ After modeling, the mice were euthanized, and the length of the whole large intestine was recorded.

### Histological Analysis

Colonic tissue sections were fixed in 4% paraformaldehyde overnight at 4 °C then paraffin was embedded, sectioned, and stained with hematoxylin and eosin. For histological scoring, slides were evaluated based on the following parameters: immune cell infiltration (1‐5), mucosal thickening/edema (1‐5), crypt length, and crypt abscess/erosion (1‐5).^[^ ^45^ ^]^

### Quantifications and Statistical Analysis

All data were expressed as the mean ± standard deviation (S.D.). The data shown in the study were obtained in at least three independent experiments performed in a parallel manner. Statistical analysis was performed using a two‐tailed Student's *t*‐test. Probability values of <0.05, 0.01, and 0.001 were considered as statistically significant and marked with “*”, “**”, and “***” in respective figures. A statistical analysis was performed using GraphPad Prism.

## Conflict of Interest

The authors declare no conflict of interest.

## Author Contributions

L.X., C.L., C.W., and Z.W. contributed equally to this work. W.Y. designed the study, analyzed data, and wrote the paper with the help of L.X., C.L., C.W., D.C., and X.Y., L.X., C.L., and C.W. performed biochemistry experiments, cell culture experiments and analyzed data; D.Z., C.L., C.W., and J.L. worked the structural experiment and data analysis for K408 mutation of SIRT1; Z.W., L.X., X.Z., and C.D. assisted in mass spectrometry experiments. Q.Z. and P.L. helped with the D^3^‐aspirin synthesis. L.X. and C.W. worked on the T‐cell analysis.

## Supporting information

Supporting Information

Supporting Information

Supporting Information

## Data Availability

The data that support the findings of this study are available from the corresponding author upon reasonable request.
