# Supplementary material for: Aspirin‐Mediated Acetylation of SIRT1 Maintains Intestinal Immune Homeostasis
Source: Adv Sci (Weinh). 2024 Mar 14;11(19):2306378. doi: 10.1002/advs.202306378 (PMC11109641; doi:10.1002/advs.202306378)
Supplement: Supplementary file 1 — Supporting Information [file ADVS-11-2306378-s003.pdf]

ADVANCED SCIENCE
Open Access

Supporting Information

for *Adv. Sci.*, DOI 10.1002/advs.202306378

Aspirin-Mediated Acetylation of SIRT1 Maintains Intestinal Immune Homeostasis

Liangguo Xie, Chaoqun Li, Chao Wang, Zhen Wu, Changchun Wang, Chunyu Chen, Xiaojian Chen, Dejian Zhou, Qiang Zhou, Ping Lu, Chen Ding, Chen-Ying Liu, Jinzhong Lin, Xumin Zhang, Xiaofei Yu and Wei Yu**

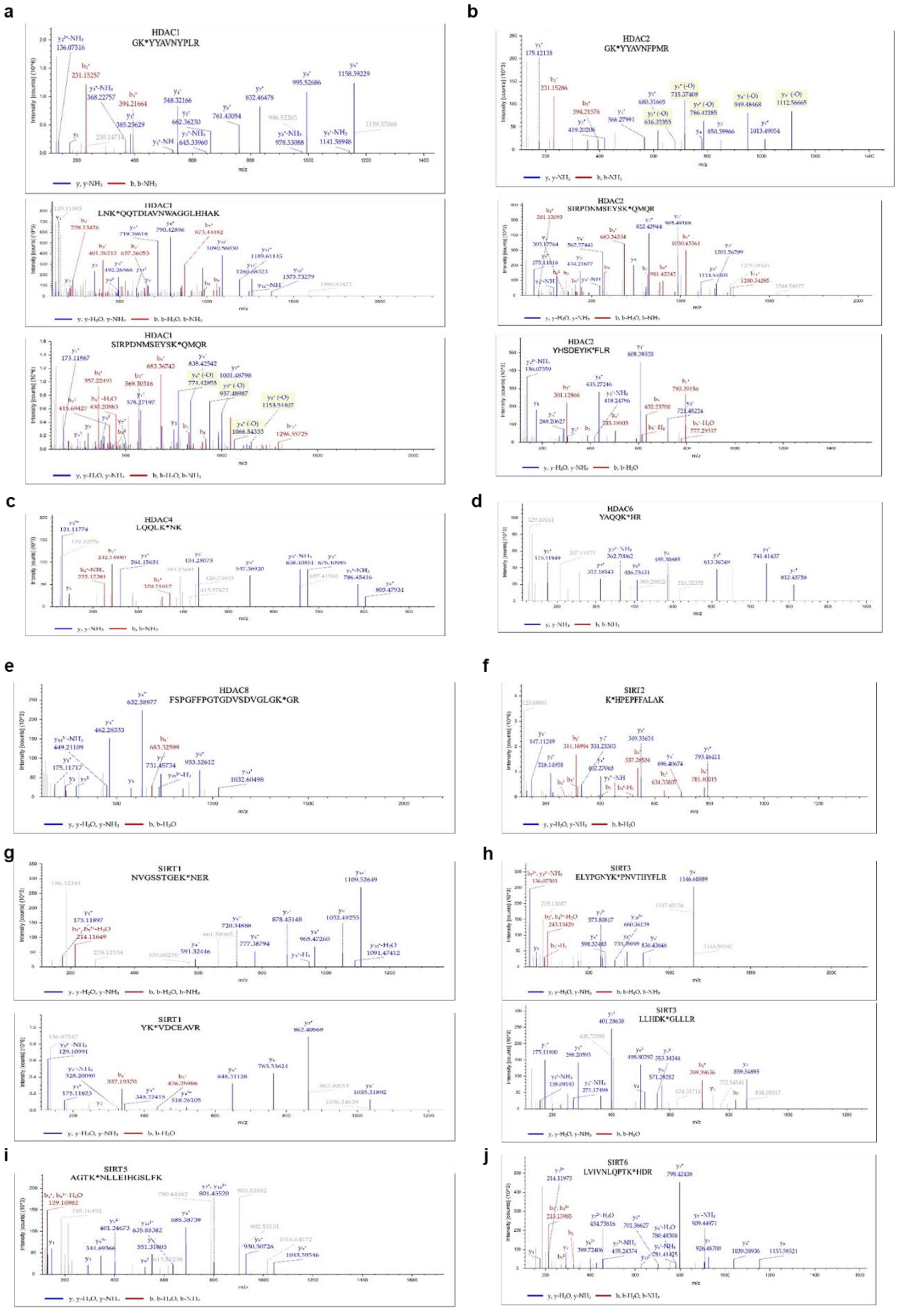

Figure S1

Figure S1

a-j, Example MS/MS spectra of aspirin-d3-mediated acetylation of HDAC family. Mass spectra of 16 D_3-acetylated peptides from 10 HDAC proteins are presented. Perspective acetylated lysine residues are marked.

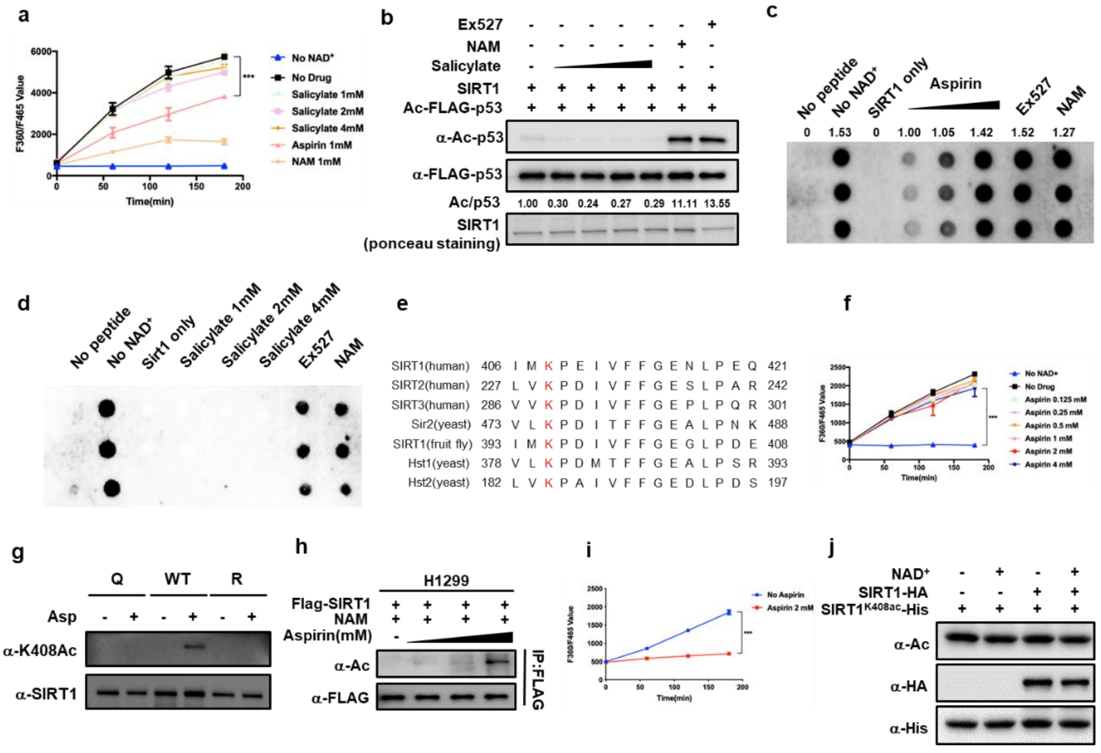

Figure S2

Figure S2

a, Effects of salicylate and NAM, EX527 on SIRT1 activity. The deacetylase activities of recombinant human SIRT1 were measured by monitoring the fluorescence intensity (excitation at 360 nm and emission at 460 nm) using a substrate peptide with one end coupled to a fluorophore and the other end to a quencher. A reaction without NAD+ was performed as a negative control. Data are presented as mean ± s.d., n = 3 wells, from three independent experiments.

b, Representative immunoblot of three independent experiments measuring SIRT1 deacetylase activities. Acetylated p53 was incubated with SIRT1 protein and indicated doses of salicylate, Ex527 and NAM for 3 h, p53 acetylation was assessed by immunoblotting with anti-p53 K382Ac antibody.

c,Representative immune dot-blot of three independent experiments measuring SIRT1 deacetylase activities. Native acetylated p53 peptide was incubated with different doses of aspirin for 3 h, then subjected to a dot blot assay using an anti-p53 K382Ac antibody.

d, Representative immune dot-blot of three independent experiments measuring SIRT1 deacetylase activities. Native acetylated p53 peptide was incubated with SIRT1 protein and indicated salicylate doses, Ex527 and NAM for 3 h, then subjected to a dot blot assay using anti-p53 K382Ac antibody.

e, Human SIRT1 K408 is conserved in the Sirtuins family. The Human SIRT1 sequence surrounding K408 was aligned with other Sirtuins or their homologs from various species.

f, Aspirin-acetylated SIRT1 K408R were assayed for SIRT1 steady-state kinetics. Effects of aspirin on SIRT1 K408R activity. Data are presented as mean ± s.d., n = 3 wells, from three independent experiments.

g, Representative immunoblot of three independent experiments characterizing acK408-SIRT1 antibody. FLAG-SIRT1 proteins were incubated with Aspirin for 2 h, the SIRT1 acetylation was assessed by immunoblotting with anti-SIRT1 K408Ac antibody.

h, Representative immunoblot of three independent experiments showing acetylated SIRT1 in H1299 cells. H1299 cells were transfected with FLAG-SIRT1 plasmids， after 24h transfection cells were treated with 0.5 mM NAM plus dose-increased aspirin and FLAG- SIRT1 acetylation was analyzed by western blot after immunoprecipitations.

i, Aspirin-acetylated SIRT1 were assayed for SIRT1 steady-state kinetics. H1299 cells were transfected with FLAG-SIRT1 plasmids, 24h after transfection, cells were treated with DMSO or aspirin. FLAG-SIRT1 deacetylase activity was examined after immunoprecipitation and normalized against the protein level. Data are presented as mean ± s.d., n = 3 wells, from three independent experiments.

j, Representative immunoblot of three independent experiments showing acetylated K408-SIRT1. *In vitro* deacetylation assay of SIRT1 K408ac by SIRT1-HA immunoprecipitated from HEK293T were performed and inhibitors(NAM: 500 μM, Ex527: 10 μM) of SIRT1 were added as a control. The purified precipitants were analyzed with anti-pan-acetyllysine,anti-His and anti-HA antibody.

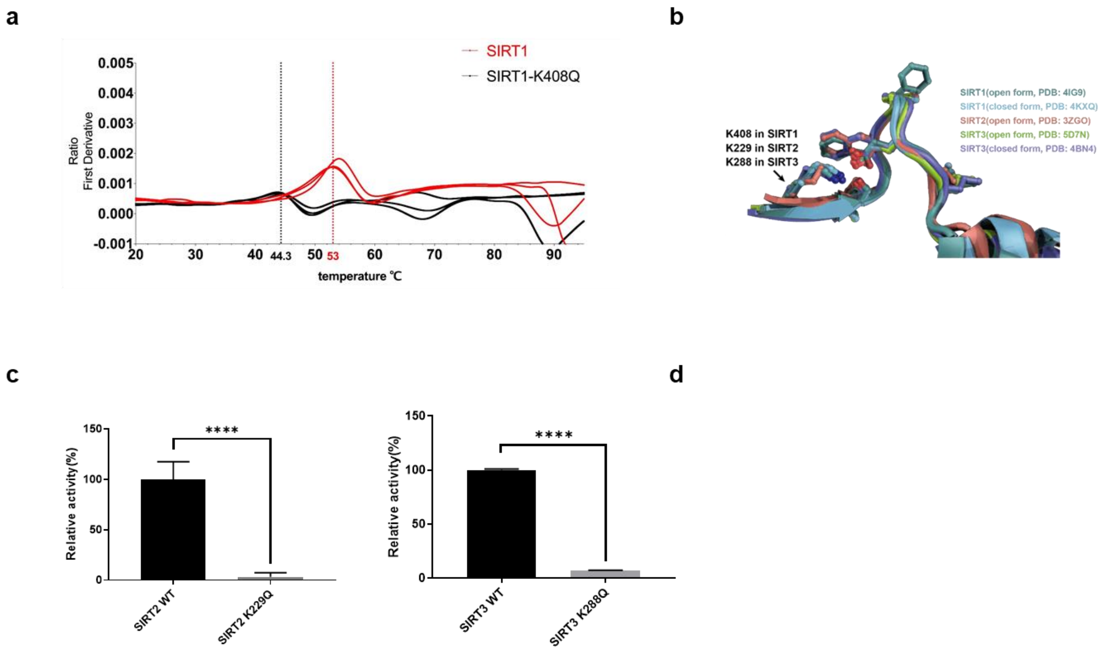

Figure S3

Figure S3

a, Melting curves for WT-SIRT1 and K408Q. Shown is the first derivative of the ratio of autofluorescence at 350 and 330 nm.

b, A superimposition of the connecting loop from structures of the Sirtuins family. The Sirtuins family structures often display two conformations: a closed conformation when an NAD+ ligand or a peptide substrate is present and an open one without any ligand or substrate-bound. In all structures of the Sirtuins family determined so far, the loop region adopts a rigid form which is stabilized by the lysine residue (K408 in SIRT1, K229 in SIRT2, and K288 in SIRT3).

c, Quantification of SIRT2 and SIRT3 activity; n=3 biologically independent samples per group, represented as the mean s.e.m.; **** P< 0.0001, two-tailed Student's t-test. *In vitro* deacetylation assay of SIRT2/3 WT and SIRT2-K229Q/SIRT3-K288Q mutant. The protein was immunoprecipitated from HEK293T expressing SIRT2/3-HA or SIRT2-K229Q/SIRT3-K288Q.

d,Relative abundance of AcK229 sites identified in SIRT2 protein from the two cell treatments.

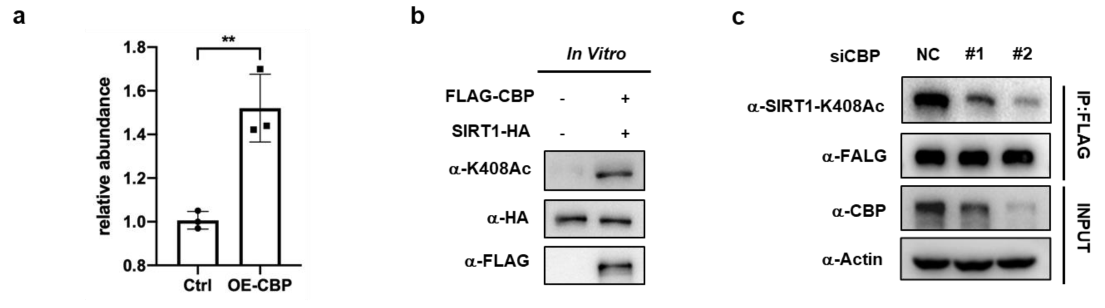

Figure S4

Figure S4

a, CBP acetylates SIRT1 in the K408 site *in vitro*. FLAG-CBP or SIRT1-HA was expressed in HEK293T cells and purified by Flag beads or HA beads. *In vitro* acetylation assay of SIRT1 K408ac by FLAG-CBPwere performed.

b.Representative immunoblotting of three independent experiments shows that the inhibition of CBP expression by small interfering RNA reduces the acetylated K408-SIRT1 level.

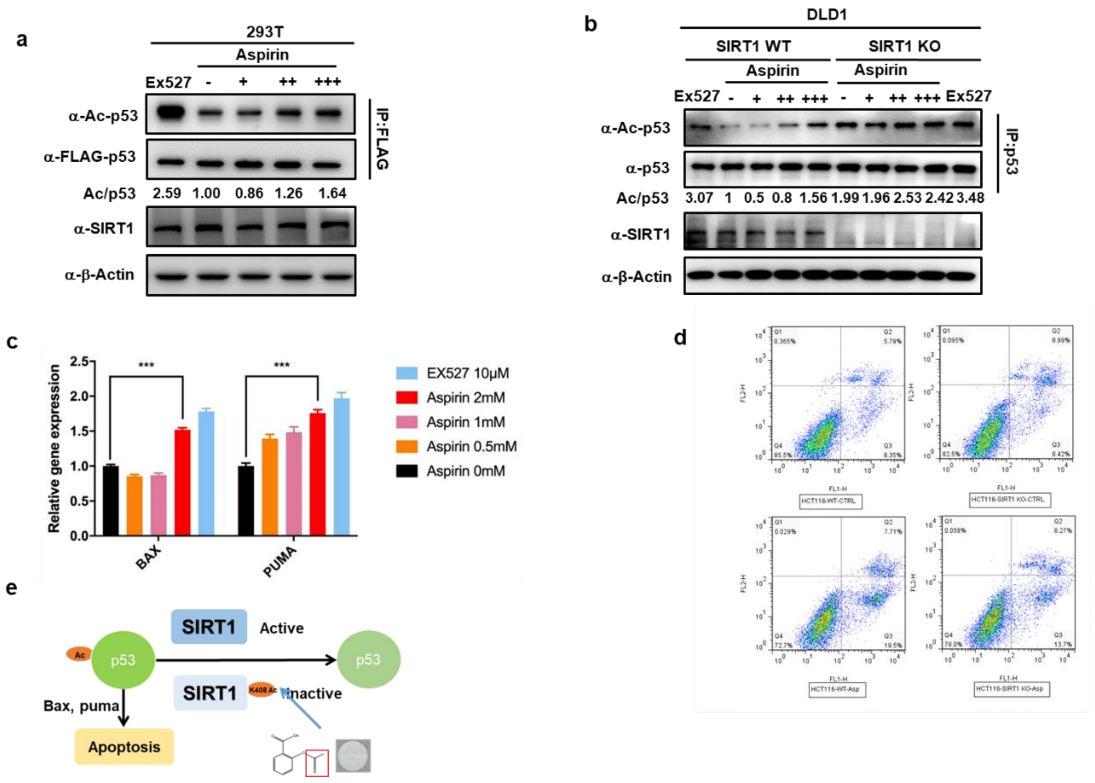

Figure S5

Figure S5

a, Representative immunoblot of three independent experiments showing the SIRT1 and its substrates p53ac in HEK293T cells. The cells were transfected with p53-FLAG and treated with the indicated doses of Aspirin for 24 h. Then the p53-FLAG was immunoprecipitated and p53 acetylation was assessed.

b, Representative immunoblot of three independent experiments showing the SIRT1 and its substrates p53ac in DLD1(SIRT1 WT) and DLD1(SIRT1 KO) cells treated with different doses of Aspirin for 24 h. p53 acetylation was assessed after p53 immunoprecipitation.

c, Relative gene expression of p53 downstream genes (BAX, PUMA) were measured in HCT116 cells treated with indicated doses of Aspirin for 24 h. Error bars represent s.d., n=3, from three independent experiments.

d, HCT116(WT) and HCT116(SIRT1 KO) cells were treated with DMSO or 2 mM aspirin, and apoptosis was measured by FACS. Error bars represent s.d., n=3, from three independent experiments.

e, The model shows that Aspirin acts as a SIRT1 inhibitor and regulates SIRT1-dependent p53 activation and cell apoptosis.
